# Drug Response Associated With and Prognostic lncRNAs Mediated by DNA Methylation and Transcription Factors in Colon Cancer

**DOI:** 10.3389/fgene.2020.554833

**Published:** 2020-11-04

**Authors:** Jiayu Zhang, Zhen Shen, Zheyu Song, Jian Luan, Yezhou Li, Tiancheng Zhao

**Affiliations:** ^1^Department of Gastrointestinal Colorectal and Anal Surgery, China-Japan Union Hospital of Jilin University, Changchun, China; ^2^Department of Vascular Surgery, China-Japan Union Hospital of Jilin University, Changchun, China; ^3^Department of Endoscopy Center, China-Japan Union Hospital of Jilin University, Changchun, China

**Keywords:** colon cancer, DNA methylation, transcription factor, lncRNA, drug response, survival

## Abstract

Colon cancer is the most commonly diagnosed malignancy and the leading cause of cancer deaths worldwide. As well as lifestyle, genetic and epigenetic changes are key factors that influence the risk of colon cancer. However, the impact of epigenetic alterations in non-coding RNAs and their consequences in colon cancer have not been fully characterized. We detected differential methylation sites (DMSs) in long non-coding RNA (lncRNA) promoters and identified lncRNA expression quantitative trait methylations (lncQTMs) by association tests. To investigate how transcription factor (TF) binding was affected by DNA methylation, we characterized the occurrence of known TFs among DMSs collected from the MEME suite. We further combined methylome and transcriptome data to construct TF–methylation–lncRNA relationships. To study the role of lncRNAs in drug response, we used pharmacological and lncRNA profiles from the Cancer Cell Line Encyclopedia (CCLE) and investigated the association between lncRNAs and drug activity. We also used combinations of TF–methylation–lncRNA relationships to stratify patient survival using a risk model. DNA methylation sites displayed global hyper-methylation in lncRNA promoters and tended to have negative relationships with the corresponding lncRNAs. Negative lncQTMs located near transcription start sites (TSSs) had more significant correlations with the corresponding lncRNAs. Some lncRNAs found to be mediated by the interplay between DNA methylation and TFs were previously identified as markers for colon cancer. We also found that the ELF1-cg05372727- LINC00460 relationship were prognostic signatures for colon cancer. These findings suggest that lncRNAs mediated by the interplay between DNA methylation and TFs are promising predictors of drug response, and that combined TF–methylation–lncRNA can serve as a prognostic signature for colon cancer.

## Introduction

Colon cancer is the most commonly diagnosed malignancy and leading cause of cancer deaths worldwide (Arnold et al., 2017). The development of colon cancer is related to lifestyle and exposure to environmental risks (Kuipers et al., 2015). An accumulation of genetic and epigenetic alterations can also facilitate the development of colon cancer from normal mucosa (Hong, 2018). Early screening of colonic lesions is beneficial for colon cancer diagnosis and can reduce mortality (Suehiro et al., 2008). DNA methylation, a stable epigenetic mark, has been shown to influence a wide range of biological mechanisms (Jin and Liu, 2018). Studies have emphasized the important role of DNA methylation in the early detection of colon cancer (Ashktorab et al., 2013, 2014). However, DNA methylation in the non-coding genome and its downstream effect on colon cancer have not been fully characterized.

Human genomes are pervasively transcribed into non-coding RNAs (Yan et al., 2015). Long non-coding RNAs (lncRNAs) are capped transcripts longer than 200 nucleotides (Derrien et al., 2012). The study of lncRNAs has broadened our understanding of many biological and disease processes (Dhanoa et al., 2018; Yarani et al., 2018); for instance, lncRNAs can influence cancer progression in multiple ways, including having effects on drug sensitivity, patient prognosis, and cancer cell inhibition (Ning et al., 2017; Zhang et al., 2017; Bermudez et al., 2019). Some previous studies found that lncRNAs could be regulated by epigenetic alterations, with an impact on cancer development (Wang et al., 2018c). Therefore, systematical identification of DNA methylations on lncRNAs and their consequences in colon cancer will be helpful for cancer prevention and treatment.

TFs are cell fate controllers that are frequently involved in many diseases, including cancers (Lambert et al., 2018). A single TF can bind to thousands of sites throughout the genome, typically by recognizing DNA sequences, to guide gene transcription activity (Spitz and Furlong, 2012). Recent studies revealed that TF binding can be affected by alterations in cytosine methylation patterns (Maurano et al., 2015). For instance, DNA methylation at CpG islands of gene promoters blocks TF binding and silences gene expression (Bird, 2002). Methylation-sensitive TFs could be involved in the DNA methylation mediated transcription process in human cancers (Wang et al., 2020). DNA methylation could affect TF-target transcriptional regulation circuits across various cancer types (Liu et al., 2019). There are also cytosine methylations that can promote TF binding (Yin et al., 2017). Based on this, we studied the interactions between DNA methylation and TFs and their role in lncRNA regulation in colon cancer.

We systematically analyzed DNA methylations in lncRNA promoters and identified lncRNAs that were mediated by the interplay between DNA methylation and TFs. The lncRNAs identified in our study were associated with drug response in colon cancer. One TF–methylation–lncRNA could be used to stratify patient prognosis in colon cancer. We hope our results will provide guidance for clinical research on colon cancer in the future.

## Materials and Methods

### Methylation and Expression Datasets

We downloaded methylation profiles from the Infinium HumanMethylation450 BeadChip and expression profiles based on RNA sequencing in colon cancer (COAD, colon adenocarcinoma) from the UCSC Xena archive (v1.0^1^, “Methylation450k” and “gene expression RNAseq” in the TCGA Colon Cancer cohort). Expression values were log_2_ transformed in UCSC Xena. In order to further explore the relationship between DNA methylation and lncRNA expression, we only kept colon samples that appeared in both the DNA methylation and the expression datasets. The resulting 306 colon cancer and 19 adjacent mucosa tissue samples were used for downstream analysis.

### Construction of Methylation Profiles in lncRNAs

We omitted methylation probes that had single-nucleotide polymorphisms near the probe sequence or that were located in sex chromosomes as annotated in the Illumina sequencing platform. We obtained lncRNA annotation files from the GENCODE website (v22, GRCh38) (Frankish et al., 2019), including long intergenic non-coding RNA, antisense, process transcript, sense intronic, 3’ overlapping non-coding RNA, sense overlapping, non-coding, and macro lncRNAs. The genomic coordinates of probes were transformed from GRCh37 to GRCh38 using the UCSC liftOver tool with the default parameters (v1.0) (Zhu et al., 2009) and further mapped into lncRNA promoters [2 kb upstream to 2 kb downstream of the corresponding transcription start sites (TSSs)] to give the methylation profile of lncRNA promoters.

### Identification of DMSs in lncRNAs

Methylation probes that had missing values in more than 80% of samples were excluded from the analysis; for other probes, missing values were imputed using the impute package in the R software with the default parameters. We used Wilcoxon rank-sum test (two-tailed) and differences in mean values to analyze the differences in methylation between colon cancer and corresponding mucosa tissue samples; *p*-values were corrected using the false discovery rate (FDR) method, probes with FDR < 0.05, and absolute methylation difference (mean methylation level in colon cancer samples—mean methylation level in normal samples) > 0.3 were regarded as DMSs. A circos plot of these sites was constructed using the circlize R package (Gu et al., 2014).

### Identification of lncQTMs

The associations between these DMSs and the corresponding lncRNAs were determined by Spearman’s rank correlation test (FDR < 0.05) (Gutierrez-Arcelus et al., 2013; Bonder et al., 2017; Husquin et al., 2018). Methylation sites in the correlated methylation–lncRNA pairs were considered to be potential epigenetic regulators of the lncRNAs; we called these methylation–lncRNA pairs lncQTMs.

### Identification of TF Binding Motifs Around lncQTMs

To explore the interactions between DNA methylation and TFs, we collected comprehensive human TF motif position weight matrices from the MEME suite (v5.1.1) (Bailey et al., 2015) and scanned motif occurrence in the regions ± 100 bp of methylation sites in lncQTMs using the FIMO software (v5.0.3, –verbosity 2 –thresh 0.0001) (Grant et al., 2011). Among the significant motifs (*p* < 0.0001), we filtered out those that did not cover the methylation sites. These motifs were expected to have more enrichment of lncQTMs than background methylation sites (all methylation sites within lncRNA promoters). For each motif, we computed the odds ratio (OR); motifs equal to or greater than 1.1 were retained for further study (Yao et al., 2015).

### Establishing the TF–Methylation–lncRNA Network

For the selected motifs, we used association tests to identify significantly correlated TF–methylation pairs (Spearman’s rank correlation test, FDR < 0.05). As TFs can regulate lncRNA expression by binding to methylation sites, we also tested the relationships between TFs and their target lncRNAs (Spearman’s rank correlation test) and retained correlated TF–lncRNA pairs (FDR < 0.05). Based on these results, we constructed a TF–methylation–lncRNA relationship network for colon cancer.

### Functional Annotation of lncRNAs Regulated by TFs and DNA Methylation

We collected RNA-RNA interaction data from RNAInter database (v1.0) (Lin et al., 2020). Next, we mapped lncRNAs and mRNA into RNA-RNA interaction data and obtained lncRNA-mRNA interactions. We tested expression correlations of lncRNA-mRNA interactions and identified significantly correlated lncRNA-mRNA pairs to form a network (Spearman’s rank correlation test, FDR < 0.05). After that, mRNAs in the network were used for functional annotation using the Enrichr (v2.0) web server (Kuleshov et al., 2016).

### Collection of Drug Response Data in Colon Cancer

The CCLE database provides comprehensive pharmacological profiles across hundreds of cell lines (Barretina et al., 2012). It also includes RNA sequencing data for cell lines, enabling quantification of lncRNAs. We downloaded drug response data from CCLE (v1.0), selecting 15 colon cancer cell lines (COAD) that were relevant to the current study. We used the activity area to evaluate drug sensitivity and kept lncRNAs that were expressed in at least 50% of cell lines.

### Exploring the Association Between Drug Response and lncRNA Expression

Spearman’s rank test was used to test the correlation between lncRNA expression and drug activity area. We divided cell lines into two groups based on median expression of the lncRNA of interest, and determined the differences in activity area between the two groups (Wilcoxon rank-sum test, two-tailed). The drug activity area was normalized using the z-score method. Cell lines with an activity area at least 0.8 standard deviations (SDs) above the mean were regarded as sensitive, and those with an activity area at least 0.8 SDs below the mean were regarded as resistant; intermediate cell lines were eliminated (Dong et al., 2015). We also determined the lncRNA expression difference between sensitive and resistant groups. Considering the small size of samples used in the drug response (*n* = 15), *p*-values were not corrected using the FDR method (Albers, 2019).

### Survival Analysis Based on TF–Methylation–lncRNA Relationship

We downloaded matched TCGA overall survival (OS) survival records from the TCGAbiolinks R package (Colaprico et al., 2016). In total, there were 292 patients that have the matched OS information in this study. We randomly divided the 292 patients into training (*n* = 146) and testing set (*n* = 146) to test the efficiency of TF–methylation lncRNA relationships in survival prediction. Firstly, in training set, we determine the associations between TFs, methylation sites, lncRNAs and patient OS by conducting univariate Cox regression model, respectively. Meanwhile, for each TF–methylation–lncRNA relationship, we applied a multivariate Cox proportional regression model to determine the associations between TF–methylation–lncRNA and patient OS in training set. Next, we designed risk score models that related with TFs, DNA methylation sites, lncRNAs and TF–methylation–lncRNA based on the coefficient from the Cox regression model, in which each colon cancer sample was assigned a risk score as follows:

TFs associated model: *risk score_i_* = β_*tf*_*TF_i_*

DNA methylation sites associated model: *risk score_i_* = β_*meth*_*Meth_i_*

LncRNAs associated model: *risk score_i_* = β_*lnc*_*lnc_i_*

TF–methylation–lncRNA associated model: *risk score_i_* = β_*tf*_*TF_i_* + β_*meth*_*Meth_i_* + β_*lnc*_*lnc_i_*

where *i* represents colon cancer sample i, β_*tf*_ is the coefficient of the TF, β_*meth*_ is the coefficient of the methylation site, β_*lnc*_ is the coefficient of the lncRNA, and *TF*_*i*_, *Meth*_*i*_ and *lnc*_*i*_ are the corresponding values for the TF, methylation site, and lncRNA, respectively, in colon cancer sample *i*.

According to the patient risk scores, we categorized colon cancer samples into two risk groups using the median risk score and applied Kaplan–Meier survival analysis (Goel et al., 2010). Differences in OS between the two groups were determined by log-rank test.

Disease free survival (DFS) records were collected from cBioPortal (v3.4.2) (Cerami et al., 2012; Gao et al., 2013); 255 patients had matched DFS records in our study. We applied the same risk model and did Kaplan–Meier survival analysis for DFS.

### Statistical Analysis

Statistical analyses were performed based on R program (v3.6.1). Statistical significance is shown as ^∗^*P* < 0.05, ^∗∗^*P* < 0.01, or ^∗∗∗^*P* < 0.001.

## Results

### lncRNA Promoters Show Global Hyper-Methylation in Colon Cancer

**FIGURE 1:**
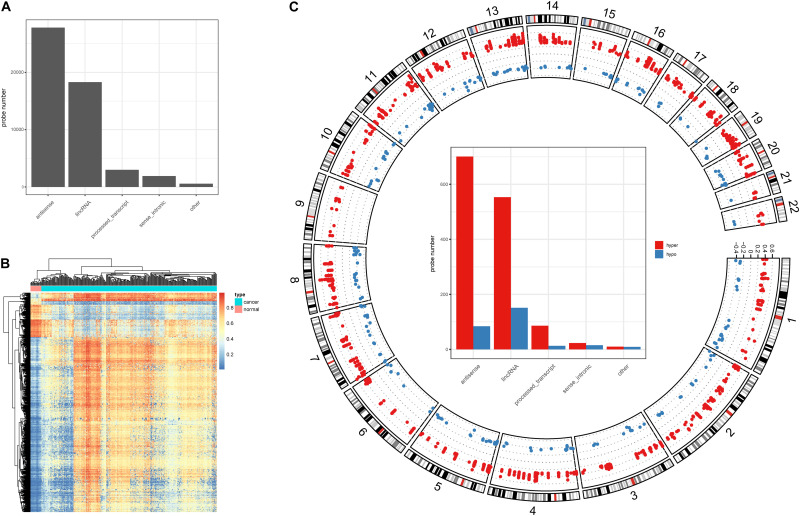
DNA methylation character in lncRNA promoters. **(A)** Probe number in each lncRNA class. **(B)** Heatmap of the DMSs in lncRNA promoters. **(C)** Circos plot of the hyper- and hypo DMSs; *y*-axis shows the methylation difference level, inside bar plot represent the hyper- and hypo DMSs statistics in each lncRNA class.

Alteration of DNA methylation has been implicated in tumor progression and disease development, and studies have identified a potential role for DNA methylation in early screening of colon cancer (Chen et al., 2019). Here, we investigated DNA methylation in lncRNA promoters in colon cancer. We successfully mapped 43,881 methylation probes to lncRNA promoter regions (Figure 1A) to study DNA methylation in lncRNAs. Next, we identified 1,566 DMSs in lncRNAs (FDR < 0.05, absolute methylation difference > 0.3, Figure 1B). Most of the DMSs displayed higher methylation values in cancer samples than in normal samples (Figure 1B). We further classified DMSs as hyper-DMSs (methylation difference > 0) or hypo-DMSs (methylation difference < 0). All chromosomes had more hyper-DMSs than hypo-DMSs, and chromosome 9 had only hyper-DMSs in lncRNA promoters (Figure 1C and Supplementary Table 1). In addition, we found many more hyper-DMSs than hypo-DMSs in antisense, lincRNA, and process transcripts (8. 3-, 3. 7-, and 6.6-fold differences, respectively, Figure 1C). These hyper- and hypo-DMSs are potential regulators of the host lncRNAs and may participate in the pathogenic processes of colon cancer.

### Relationships Between Promoter Methylation and lncRNA in Colon Cancer

**FIGURE 2:**
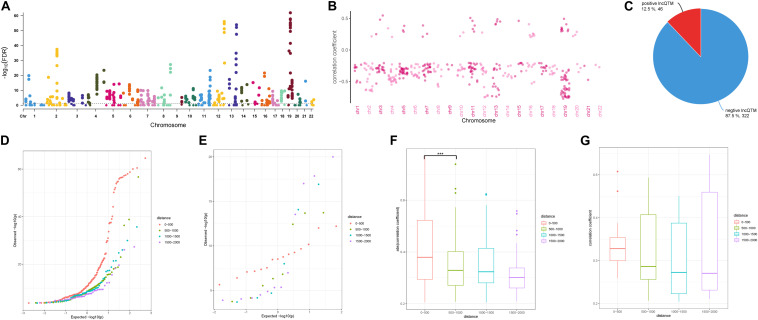
Character of lncQTMs in colon cancer. **(A)** Manhattan plot methylation-lncRNA FDR values in each chromosome (Spearman’s rank correlation test). The horizontal dotted line indicates the significant cut-off (FDR = 0.05) of lncQTMs. **(B)** Distribution of correlation coefficient of lncQTMs in each chromosome. **(C)** The number of positive and negative LncQTMs. **(D)** Quantile-Quantile Plot of negative lncQTMs for each distance category. **(E)** Quantile-Quantile Plot of positive lncQTMs for each distance category. **(F)** Boxplot of negative lncQTMs correlation coefficient relative to different distance. **(G)** Boxplot of positive lncQTMs correlation coefficient relative to different distance. ***Denote p < 0.001.

To investigate the role of the identified DMSs in lncRNA transcription regulation, we systematically identified correlated DNA methylation site–lncRNA pairs (lncQTMs) in colon cancer (Figure 2A). In most chromosomes, promoter methylation sites were more likely to negatively regulate lncRNA expression; indeed, some chromosomes had no positive lncQTM pairs (Figure 2B). In total, there were 322 negative lncQTMs (correlation coefficient < 0, 87.5%) and 46 positive lncQTMs (correlation coefficient > 0, 12.5%) (Figure 2C). This indicates that promoter methylation sites are more likely to inhibit lncRNA expression in colon cancer, consistent with previous findings about DNA methylation in regulation of protein-coding genes (Schelleckes et al., 2017). In addition, we classified both positive and negative lncQTMs according to the distance between methylation sites and TSSs. For negative lncQTMs, methylation sites closer to lncRNA TSSs displayed a more significant character (smaller *p*-value, Figure 2D), however, for positive lncQTMs, some methylation sites located within 1,500–2,000 bp of lncRNA TSSs showed more significant character than methylation sites within 200 bp (Figure 2E). Furthermore, we analyzed correlation strengths for the lncQTMs in different distance classes: in negative lncQTMs, DNA methylation sites located within 500 bp of TSSs showed strong correlations with lncRNAs (two-tailed Wilcoxon rank-sum test, FDR < 0.05), and there was no significant difference when the distance increased (Figure 2F). For positive lncQTMs, they did not show significant differences when the distance increased (Figure 2G). This suggests that lncRNA promotor methylation sites are likely to inhibit expression, and that sites located closer to the TSSs have stronger correlations with the corresponding lncRNAs.

### Identification of TFs Around lncQTMs in Colon Cancer

**FIGURE 3:**
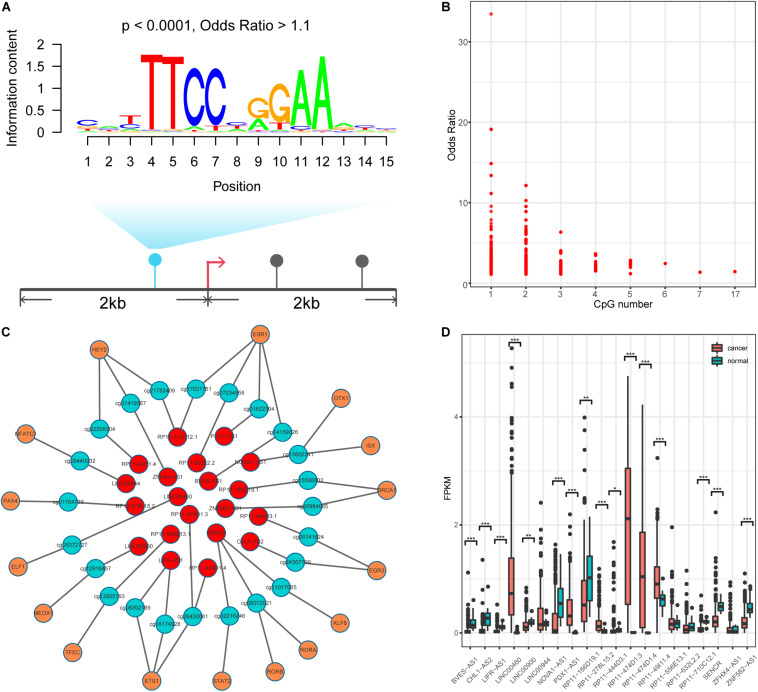
Identification TF-methylation-lncRNA relationships. **(A)** An example for illustrating the TF motif occurrence in lncQTM. **(B)** Lower OR value and the related CpG number. **(C)** An unweighted network of TF-methylation-lncRNA relationships, including 59 nodes (16 TFs, 23 methylation sites, and 20 lncRNAs), 49 edges (25 edges between TFs and DNA methylation sites, 24 edges between DNA methylation sites and lncRNAs). Red nodes represent lncRNAs, green nodes represent methylation sites, and orange nodes represent TFs. **(D)** LncRNA expression level for colon cancer and normal samples (Wilcoxon rank-sum test, two-tailed). *Denote *p* < 0.05, **denote *p* < 0.01, ***denote *p* < 0.001.

**FIGURE 4:**
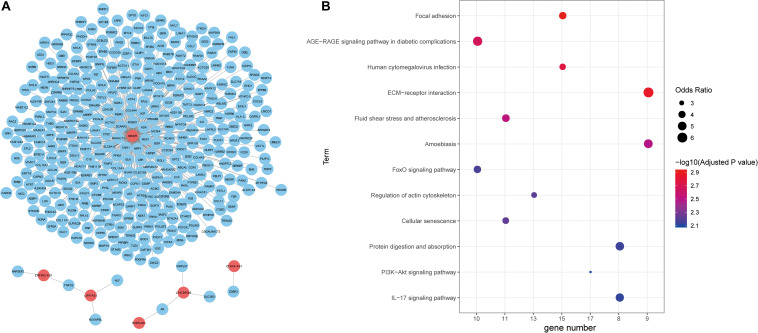
Functional annotation of lncRNAs. **(A)** The unweighted network of lncRNA-mRNA pairs, including 370 nodes (6 lncRNAs and 364 mRNAs), 366 edges. Red nodes represent lncRNAs, blue nodes represent mRNAs. **(B)** Significant KEGG pathways derived from the Enrichr web server.

DNA methylation can modulate gene expression in multiple ways (Moore et al., 2013); specifically, it can shape TF binding events across human tissues (Hu et al., 2013; O’Malley et al., 2016). We thus speculated that human TFs might be sensitive to changes in DNA methylation in lncQTMs. Based on this speculation, we scanned TF motif occurrence in lncQTMs using the FIMO tool (Figure 3A). We expected the significant TF motifs (*p* < 0.0001, methylation site located within the motif) to be more enriched in lncQTMs than in all lncRNA promoter methylation sites (OR > 1.1). After filtering, we obtained 431 motifs located in lncQTMs (Figure 3B). These motifs associated with TFs are potential regulators of the corresponding lncRNAs. To confirm the relationships among TFs, methylation sites, and lncRNAs, we identified significantly correlated TF–methylation site and TF–lncRNA pairs using Spearman’s rank test (FDR < 0.05). Finally, we obtained 26 TF–methylation–lncRNA relationships in colon cancer, comprising 16 TFs, 23 methylation sites, and 20 lncRNAs (Figure 3C). DNA methylation on gene promoters generally repress transcription, whereas some researchers had reported the role of DNA methylation in promoting the transcription (Harris et al., 2018). Among the network, there were 24 methylation-lncRNA pairs; 21 pairs were negatively correlated while three pairs were positively correlated. Therefore, the hyper-DMSs in colon cancer may lead to the loss of TF-lncRNA relationship when they are negatively correlated with the corresponding lncRNAs. For the hypo-DMSs in colon cancer, they may lead to the gain of TF-lncRNA relationship when they are negatively correlated with the corresponding lncRNAs. Positively correlated pairs may have the opposite relationship with the negatively correlated pairs. Thus, these DMSs in network may lead to the gain of seven TF-lncRNA regulation relationships, and the loss of 19 TF-lncRNA regulation relationships. The regulatory relationship among TFs, DNA methylation sites, and lncRNAs is complex, and our results need to be further validated. Of the 20 lncRNAs regulated by the interplay between TF and methylation, 16 lncRNAs displayed a differential expression pattern (two-tailed Wilcoxon rank-sum test, *p* < 0.05, Figure 4D) between cancer and normal samples. These included lncRNA LINC00944, which has been shown to participate in the process of liver metastasis in colorectal cancer (Li et al., 2020), and lncRNA LINC00460, hypo-methylation of which can promote colon cancer and represents a potential biomarker (Zhang et al., 2019). Therefore, these TF–methylation–lncRNA events may modulate colon cancer progression and altering the interactions among them may be beneficial for therapy and prognosis.

Next, we explored the function of lncRNAs that were regulated by the interplay between TFs and DNA methylation. By integrating RNA-RNA interaction and gene expression profiles, we established a lncRNA-mRNA network (Figure 4A). Noticeably, lncRNA SENCR could interact with 356 mRNA in colon cancer. Previous studies had reported the role of SENCR in multiple human cancers (Jiang et al., 2017; Xu et al., 2019; Zheng et al., 2019). DANCR had also been proven to promote the proliferation and metastasis of colorectal cancer via miR-577 sponging (Wang et al., 2018a). By conducting functional enrichment analysis, we observed some pathways had been elaborated to play vital roles in colon cancer (Figure 4B). For example, cellular senescence could predict the treatment outcome for metastasized colorectal cancer (Haugstetter et al., 2010). Interleukin-17 (IL-17) acts as an important promoter to affect the initiation and progression of colorectal cancer (Wu et al., 2013). Collectively, functional enrichment results showed lncRNAs in network potentially affect colon cancer by interacting with the corresponding mRNAs.

### lncRNAs Are Associated With Drug Response in Colon Cancer

**FIGURE 5:**
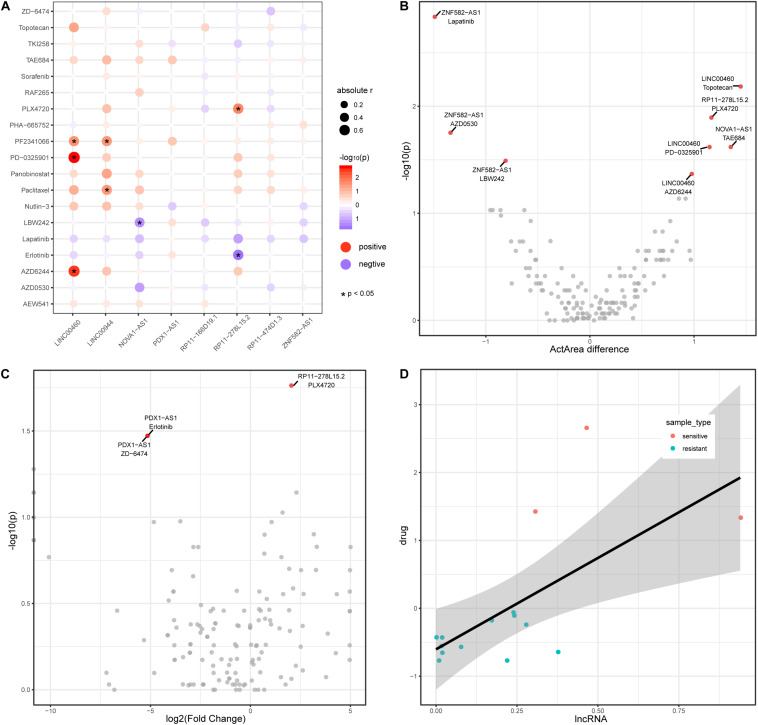
LncRNAs are associated with drug activity in colon cancer. **(A)** Correlation between lncRNAs (*x*-axis) and drug activity (*y*-axis, activity area) by Spearman’s rank correlation test. Circle size represents the absolute correlation coefficient between lncRNAs and drugs, red indicates positive correlation, and blue indicates negative correlation. *Denote *p* < 0.05. **(B)** Scatter plot of Activity area difference (*x*-axis) and –log10(*p-*value) derived from Wilcoxon rank-sum test (two-tailed, *y*-axis) for each lncRNA-drug pair. **(C)** Scatter plot of log2(Fold Change) of lncRNA expression level between cancer and normal samples and -log10(*p-*value) derived from Wilcoxon rank-sum test (two-tailed, *y-*axis) for each lncRNA-drug pair. **(D)** Scatter plot of the expression between lncRNA RP11-278L15.2 and the activity of drug PLX4720.

Recently, lncRNAs have been used to shed light on drug responses in human cancer (Wang et al., 2018b). Here, we also investigate the associations between lncRNA and anticancer drug response. We downloaded pharmacological and lncRNA profiles for colon cancer from the CCLE database and obtained a total of 19 drug components used in colon cancer. Among the colon cancer cell lines in CCLE, eight lncRNAs (from the TF–methylation–lncRNA network) are expressed in 50% of cell lines. The expression level of four lncRNAs are positively or negatively correlated with the activity of seven drugs in colon cancer cell lines (Figure 5A), suggesting lncRNAs could reflect the drug response in colon cancer. We divided cancer samples into two groups based on the median lncRNA expression value and investigated the differences in drug response (activity area) between the two groups. Of the correlated lncRNA-drug pairs, three lncRNAs could distinguish the responses of three drugs (LINC00460 and AZD6244, LINC00460 and PD-0325901, and RP11-278L15.2 and PLX4720) in the cancer samples (Figure 5B, Wilcoxon rank-sum test, two tailed, *p* < 0.05). Next, we further stratified colon cancer samples into resistant and sensitive groups for each drug (Supplementary Table 2) and tested the lncRNA expression difference between two groups (methods). Noticeably, lncRNA RP11-278L15.2 showed significantly different expression levels between the resistant and sensitive groups of the drug PLX4720 (Figure 5C). In addition, the expression level of RP11-278L15.2 was positively correlated with the activity of drug PLX4720 (Figure 5D, *p* = 0.02, correlation coefficient = 0.59). Therefore, RP11-278L15.2 could potentially serve as a biomarker to estimate the PLX4720 effect in clinical research.

### Mining TF–Methylation–lncRNA Prognostic Signatures in Colon Cancer

**FIGURE 6:**
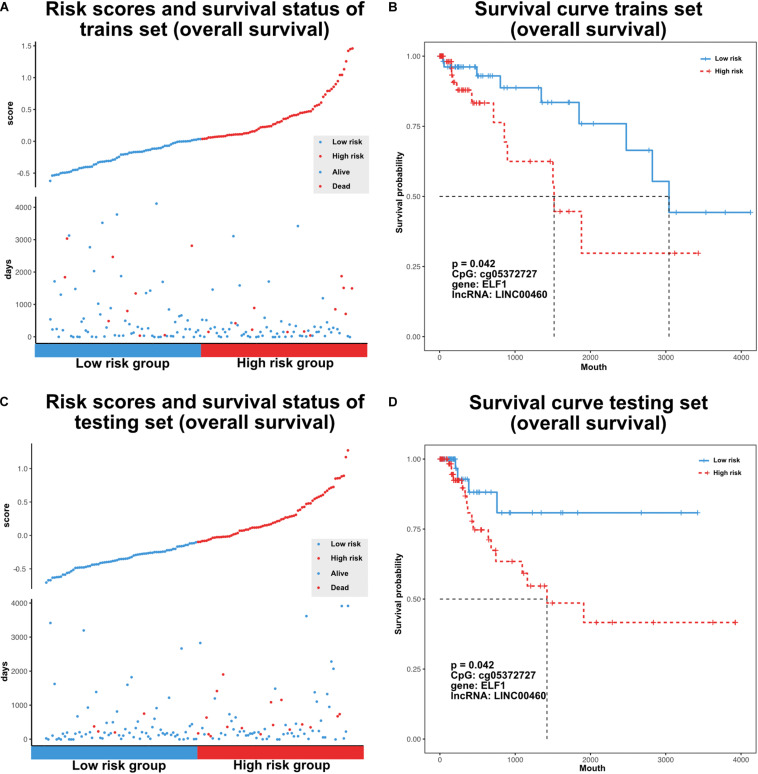
Characterize prognostic related TF-methylation-lncRNA in colon cancer. **(A)** Scatter plot of risk scores and OS status in TCGA training set. **(B)** OS curve for two risk groups in TCGA training set, *p*-value is calculated from log-rank test. **(C)** Scatter plot of risk scores and OS status in TCGA testing set. **(D)** OS curve for two risk groups in TCGA testing set, *p-*value is calculated from log-rank test.

The TF–methylation–lncRNA regulatory events detected in our research might affect patient prognosis in colon cancer. In the training set, we designed risk models related with the individual TFs, DNA methylation sites, lncRNAs, and the combination of TF–methylation–lncRNA relationship by Cox proportional regression method (methods). Patients were divided into low- and high-groups based on the obtained risk score. Next, we performed survival analysis using the Kaplan–Meier estimate method and obtained the corresponding *p*-value (log rank test). Results showed TF–methylation–lncRNA had better efficiency (lower *p* values) than individual components (Supplementary Figure 1A). Therefore, we used the risk model derived from TF–methylation–lncRNA relationships. One of the identified TF–methylation–lncRNA relationships (ELF1-cg05372727- LINC00460) was significantly associated with survival in the training set (log rank *p* < 0.05, Figures 6A,B) and could also be validated in the testing set (log rank *p* < 0.05, Figures 6C,D and Supplementary Table 3), which is the potential prognostic biomarker for colon cancer. We applied our risk model in DFS and divided patients into low- and high-groups. Results showed our risk model is not applicable for DFS (Supplementary Figure 1B and Supplementary Table 3). This result emphasizes the important role of TF–methylation–lncRNA in OS survival for colon cancer.

## Discussion

Colon cancer remains one of the most commonly diagnosed cancers worldwide. An accumulation of genetic and epigenetic alterations can result in the progression of colon cancer (Fadda et al., 2018). DNA methylation is a stable epigenetic marker, and its alteration has been shown to influence a range of diseases, including cancer (Kim and Costello, 2017). Some studies have reported the vital role of DNA methylation in protein-coding genes (Gonzalo et al., 2010; Ashktorab et al., 2013, 2014), however, its impact in non-coding genomes has not been widely understood. Here, we systematically characterized DMSs associated with lncRNAs and found that most DMSs in lncRNA promoters displayed a hyper-methylation pattern. These DMSs might modulate the lncRNA transcription process and further affect downstream biological responses triggered by the corresponding lncRNAs.

To gain insight into the relationships between DMSs and lncRNAs, we identified lncQTMs by association analysis in colon cancer. Of the 368 lncQTMs, 322 (87.5%) showed negative regulation. This suggests that DNA methylation in lncRNA promoters is likely to repress lncRNA expression in colon cancer. Negative lncQTMs that were closer to lncRNA TSSs had a greater and more significant impact on the expression level. DNA methylation alterations can influence chromatin status and TF binding events. Sequence-specific TFs can recognize typical DNA sequence (motifs) in regulatory elements and regulate the corresponding genes (Stormo, 2013). We thus made use of the known TF motifs and investigated motif occurrence around lncQTMs. We also considered the correlations among TFs, methylation sites, and lncRNAs to establish TF–methylation–lncRNA relationships. Of the lncRNAs that were regulated by the interplay between DNA methylation and TFs, lncRNAs HAND2-AS1 and LINC00460 have been shown to play a part in colon cancer development. Thus, regulation of lncRNA transcription by abnormal DNA methylation sites has a potential role in colon cancer progression.

lncRNAs have multiple functions in cancer drug sensitivity and patient prognosis (Tian et al., 2018; Jiang et al., 2019; Zhao et al., 2019). We thus investigated whether the lncRNAs shown to be mediated by DNA methylation and TFs in our study had an impact on drug efficacy and prognosis in colon cancer. Based on pharmacological and lncRNA profiles from the CCLE database, we observed that some lncRNAs are significantly correlated with drug activity. Colon cancer cell lines showed significant differences in drug response when stratified by the expression levels of lncRNAs. We divided samples into sensitive and resistant groups and found lncRNA RP11-278L15.2 display significant expression differences between the two groups of drug PLX4720, which potentially reflects the drug response of PLX4720 in clinical research. However, due to the limited cell lines analyzed in this study, we could not accurately evaluate the lncRNA efficiency in drug response prediction. With the increasement of datasets, we will continue to explore it.

We also performed survival analysis by combining TF–methylation–lncRNA relationships and found ELF1-cg05372727-LINC00460 could be used to categorize colon cancer patients into low- and high-risk groups. Furthermore, we tend to validate the prognostic efficiency of ELF1-cg05372727-LINC00460 in the independent datasets. However, there is no other public datasets that contained DNA methylation, gene expression, and OS data. By using the Gene Expression Omnibus (GEO) database, we did survival analysis based on the risk score model on two datasets that only contained expression and OS time (GSE17536, GSE59582). Results showed ELF1-LINC00460 was not associated with OS time in these two datasets (Supplementary Figures 1C,D, *p* = 0.26 in GSE17536, *p* = 0.1 in GSE59582). This suggests the importance of combining DNA methylation level of cg05372727 and the expression level of ELF1 and LINC00460 in survival prediction. With the generation of the appropriate data, we will continue to validate it.

In summary, our results provide a comprehensive view of DNA methylation in lncRNA promoters, and further demonstrate that the lncRNA transcription process is mediated by the interplay between DNA methylation and TFs. In addition, we highlight the roles of lncRNAs in drug response and patient prognosis. However, further studies are required to investigate the downstream biological mechanisms in colon cancer regulated by the lncRNAs identified in our study.

## Data Availability Statement

The public datasets can be downloaded from UCSC Xena archive (https://xenabrowser.net/datapages/), TCGA (https://portal.gdc.cancer.gov/), cBioPortal (https://www. cbioportal.org/), GEO (https://www.ncbi.nlm.nih.gov/geo/). All other data presented in this study are included in the article/Supplementary Table.

## Author Contributions

TZ and YL conceived and designed the experiments. JZ conducted most of the experiments. ZSh, ZSo, and JL give advices and supervised some experiments. JZ and ZSh wrote this manuscript. All authors read and approved the final manuscript.

## Conflict of Interest

The authors declare that the research was conducted in the absence of any commercial or financial relationships that could be construed as a potential conflict of interest.

## Footnotes

1 https://xenabrowser.net/datapages/

## Supplementary Material

Supplementary Figure 1 Exploring the association between TF-methylation-lncRNA and OS in colon cancer. **(A)** Box plots of lncRNAs, DNA methylation sites, lncRNAs, TF-methylation-lncRNA associated *p*-values (−log10 transformed, log-rank test). **(B)** DFS curve for two risk groups in TCGA colon cancer, *p-*value is calculated from log-rank test. **(C)** OS curve for two risk groups in GSE17536 dataset, *p-*value is calculated from log-rank test. **(D)** OS curve for two risk groups in GSE39582 dataset, *p*-value is calculated from log-rank test.

## Abbreviations

DMSs: differential methylation sites
lncRNA: long non-coding RNA
lncQTMs: lncRNA expression quantitative trait methylations
TF: transcription factor
CCLE: Cancer Cell Line Encyclopedia
COAD: colon adenocarcinoma
OR: odds ratio
OS: overall survival
DFS: Disease free survival
GEO: Gene Expression Omnibus.
