# Supplementary figures and images for: Drug Response Associated With and Prognostic lncRNAs Mediated by DNA Methylation and Transcription Factors in Colon Cancer
Source: Front Genet. 2020 Nov 4;11:554833. doi: 10.3389/fgene.2020.554833 (PMC7673839; doi:10.3389/fgene.2020.554833)

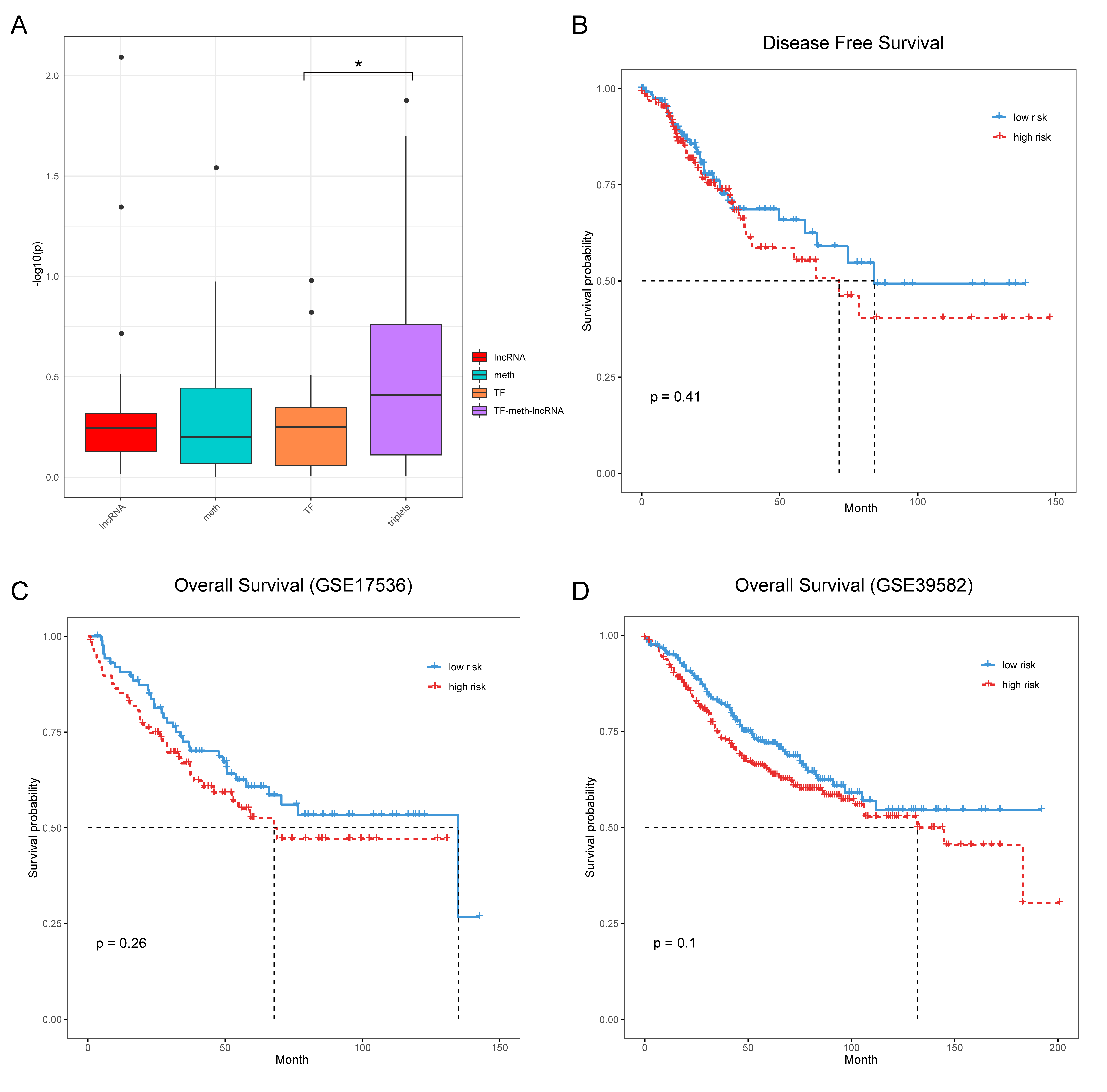

Supplement: Supplementary Figure 1 — Exploring the association between TF-methylation-lncRNA and OS in colon cancer. (A) Box plots of lncRNAs, DNA methylation sites, lncRNAs, TF-methylation-lncRNA associated p-values (−log10 transformed, log-rank test). (B) DFS curve for two risk groups in TCGA colon cancer, p-value is calculated from log-rank test. (C) OS curve for two risk groups in GSE17536 dataset, p-value is calculated from log-rank test. (D) OS curve for two risk groups in GSE39582 dataset, p-value is calculated from log-rank test. [file Image_1.TIF]
